# Strategic Development of the Genetic Counselor Profession in Germany, Austria, and Switzerland: The Establishment of the GfH Genetic Counselor Commission

**DOI:** 10.1002/jgc4.70272

**Published:** 2026-07-28

**Authors:** Gunda Schwaninger, Simone Heidemann, Kathrin Taxer, Sabine Rudnik‐Schöneborn, Johannes Zschocke

**Affiliations:** ^1^ Institute of Human Genetics Medical University Innsbruck Innsbruck Austria; ^2^ Institute of Tumor Genetics North Kiel Germany

**Keywords:** D‐A‐CH, genetic counselor, Genetische Fachberater, German Society of Human Genetics (GfH)

## Abstract

In June 2024, the German Society of Human Genetics (GfH) established a commission for Genetic Counselors (German translation: Genetische Fachberater:innen) with 86% members' approval, marking a significant step for the genetic counselor profession in Germany, Austria, and Switzerland (D‐A‐CH). This commission aims to advance the genetic counselor profession, influence policy, and drive improvements in the field. An interactive process was conducted in an online workshop format to prioritize the commission's key perspectives and goals, involving 85 participants from genetic centers across the D‐A‐CH area, including a comprehensive representation of genetic counselors and medical geneticists. The workshop event comprised introductions of speakers, commission members, presentations, and open discussions engaging participants in a structured process to identify key priorities for future initiatives. The strategic goals were developed in an interactive process involving the workshop participants. The GfH genetic counselor commission's primary goal is to create the prerequisites for the legal recognition and effective integration of genetic counselors into clinical genetic services in the D‐A‐CH region. Responding to the increasing need for more trained genetics professionals, standardized education aligned with the European core curriculum and internationally defined responsibilities and competencies for genetic counselors were defined. Among these objectives, the highest priority was assigned to the development of a scope of practice for genetic counselors in consensus with all genetics societies in the D‐A‐CH area. This interactive approach identified the most critical requirements for the full integration of genetic counselors into medical genetic services across the D‐A‐CH countries, providing a robust framework for future development and implementation of the genetic counselor profession and a prioritized development of a scope of practice for the region.

## Abstract (highlights)

**What Is Known About the Topic:**

While the profession of Genetic Counselors (GCs) has achieved recognition across Europe and worldwide, there remain several European nations in which GCs have not been integrated into the public healthcare systems. Notably, this group includes Germany, Austria, and parts of Switzerland.

**What This Paper Adds to the Topic:**

Germany, Austria, and parts of Switzerland have not yet fully integrated the genetic counseling profession. In June 2024, the German Society of Human Genetics (GfH) founded a commission to promote the professional role of genetic counselors (GCs) within the D‐A‐CH region, aiming to improve patient access to genetic services by integrating GCs into existing clinical genetics frameworks. Shortly after the commission's formation, an interactive process was initiated, engaging 85 genetics healthcare professionals from 31 genetic centers across Germany, Austria, and Switzerland. This process identified the key priorities for the full integration of GCs into medical genetic services within the D‐A‐CH countries. The results provide a strong foundation for advancing the development and implementation of the GC profession and for prioritizing the creation of a regional scope of practice.

## 1 Introduction

In June 2024, the members of the German Society of Human Genetics (GfH) voted for the establishment of a new GfH commission for genetic counselors (GCs, German translation: Genetische Fachberater:innen (GFB)), marking a significant milestone for the full integration of the GC profession in Germany, Austria, and Switzerland (D‐A‐CH countries). Following the first cohort of genetic counselors graduating from the MSc in Genetic and Genomic Counseling at the Medical University of Innsbruck in 2022, formal representation was needed. Given that the GfH board had made the establishment of the GC profession an official strategic priority for 2022 and 2023, the inaugural GCs decided to pursue a commission within the GfH rather than create a separate association. An approach that was welcomed by the GfH.

A commission is a subcommittee within the GfH that has a defined objective. In this case, establishing the professional profile of GCs within the D‐A‐CH region to enhance multiprofessional teamwork within the genetic discipline, as well as patient access to genetic services by integrating GCs into existing clinical genetics workflows. The GfH general assembly met in Berlin during the 2024 ESHG conference and voted 86% in favor of the commission, 3.9% opposed, and 10.1% abstained in an anonymous voting process. The eight members of the GfH GC commission were recruited by invitation and represent all three D‐A‐CH countries as well as all three genetic professional groups. First, all existing GCs were asked whether they wished to join the commission, resulting in five members. The Association Suisse des Conseillers en Génétique (ASCG) was invited to nominate one member. Furthermore, two medical geneticists (MGs) were invited—one a co‐founder of the Innsbruck MSc program and the other one of the first GC placement supervisors. The commission's speaker, who is both a GC and a clinical laboratory geneticist (CLG), was selected for her many years of experience heading the GfH CLG commission. The primary objective was to promote discussion on the future perspectives and aims of the GC profession in D‐A‐CH, with the goal to foster fast integration of GCs into clinical genetic teams. Here we summarize the conclusions of the interactive discussion‐based process and the resulting goals for the GC commission.

### 1.1 Workshop Participation and Structure

Following the commission's formation, an online workshop was broadly announced via the newsletters of the GfH, the Austrian Society of Human Genetics (ÖGH), and through the office of the Swiss Federal Commission for Human Genetic Testing (GUMEK); in addition, the first author issued approximately 80 personal invitations to relevant stakeholders. Since participants were required to register for the workshop, the first author communicated with all speakers and invitees, creating a preliminary list of attendees. During the online event, two members of the organizing team tracked and documented participation. A total of 85 individuals representing 31 university and privately managed genetic centers from Germany, Austria, and Switzerland participated in the online workshop. Participants included 31 MGs, one senior German GC working in the UK, 16 GCs from the D‐A‐CH region, 10 GC students from the Innsbruck MSc program in Genetic and Genomic Counseling, 22 prospective GC students, and five participants from other related professions. The workshop agenda was conceptualized by commission members and the Innsbruck MSc program team to provide a structured foundation for consensus‐building, combining a lecture‐style session encompassing five presentations with an interactive segment to identify priorities for the commission. Topics were selected to align with the commission's predefined aims and to provide essential context for integration efforts. Session 1 focused on introducing the commission, GC education and international networks, the degree of GC integration in Switzerland, the advanced development of the profession in the United States, and concepts for billing and remuneration, followed by Session 2's interactive segment to discuss and prioritize tasks for the profession that needed to be addressed by the GC commission (see program in Figure S1).

### 1.2 Workshop Methods

The workshop had the goal to identify the most critical requirements for the full integration of the GC profession into medical genetic services across the German speaking D‐A‐CH region. The Innsbruck MSc program team conceptualized, moderated, and facilitated the workshop together with GC commission members. The presentation session followed a traditional lecture‐style approach, with invited speakers covering a broad range of GC‐related topics, providing the foundational basis for the discussion. This was then followed by an interactive segment, allowing all participants to share questions and opinions. The interactive segment was conducted as a moderated plenary discussion to collect priorities for the commission. Facilitators synthesized contributions in real time with interim summaries and confirmed shared understanding on priorities before documentation. Students of the MSc program created written reports of all presentations, verbal and chat comments, questions, and the moderated discussion for later analysis of the content and the issues most present in the discussions.

At the beginning of the first session, participants were informed that the presentations and discussions would be recorded, summarized and analyzed for use in an article. Both the presentations and discussions were documented in a written report following a set structure, with only the presentations being video recorded for future analysis. The decision was made to refrain from recording the discussions to foster a more open and free exchange of opinions.

### 1.3 Session 1: Training and International Networking of Genetic Counselors in D‐A‐CH

Presentations by GCs and MGs from Germany, Austria, and Switzerland (see program in Figure S1).

#### 1.3.1 Introducing the GfH Commission for Genetic Counselors

The speaker of the GfH GC commission introduced the specialized committee dedicated to the integration of GCs in the D‐A‐CH region. The commission operates under bylaws (Statuten) jointly developed by the first active German‐speaking genetic counselors and the GfH board; these bylaws define the commission's mandate, membership composition and structure as well as the aims of the GC commission (see Textbox 1). By mutual agreement of these GCs, the GfH board, and the BVDH (Association of German Geneticists; Berufsverband der Deutschen Humangenetiker e. V.), the German‐language professional title “Genetische Fachberater:innen” was selected. The verbatim translation of genetic counselor in German is Genetische Berater:in, the prefix ‘Fach’ means ‘specialization’ and was added in coherence to the titles of CLGs—Fachhumangenetiker:in and MGs—Fachärzt:in für Medizinische Genetik (A and CH)/Fachärzt:in für Humangenetik (D).

TEXTBOX 1 **Aims of the GfH GC commission integrated in its bylaws.**

Leading initiatives to work with the GfH board to create the conditions for the legal recognition of the new academic profession of GCs in the D‐A‐CH countries, defining a scope of practice (SoP), and promoting its integration into existing clinical genetic services.

Developing strategies for the billing and remuneration of GCs in close collaboration with the BVDH.

Representing the profession of GCs both within and outside of the GfH. Promoting and coordinating collaboration with national and international professional societies and interest groups for GCs; particularly the Austrian Society of Human Genetics (ÖHG), Swiss Society of Medical Genetics (SGMG), and the Association Suisse des Conseillers en Génétique (ASCG) as well as the European Board of Medical Genetics (EBMG) as the official registration body for European GCs.

Advancing the profession in GC education, continuing professional development, supervision, and research.

In summary, the GfH GC commission is dedicated to advancing genetic counseling in the D‐A‐CH region, shaping policy, and driving improvements for the profession. Besides the GfH GC commission, German‐speaking GCs also have a representative in the European Board of Medical Genetics (EBMG) and the Transnational Alliance for Genetic Counseling board of directors to engage with GCs internationally and learn from their longstanding experience.

#### 1.3.2 Education and International Networks of Genetic Counselors

The first MSc program in Genetic and Genomic Counseling in the D‐A‐CH region was established at the Medical University in Innsbruck in 2019. It introduced the MSc curriculum in which most GCs working in the German‐speaking realm have been educated so far. For the French‐speaking part of Switzerland, education of currently active GCs was provided by the French GC programs but also by the Universities of Cardiff and Siena. All listed programs are EBMG accredited. Accordingly, the MSc held by graduates is equivalent to the internationally recognized GC qualification as it follows the standardized European core curriculum set out by the EBMG (Skirton et al. 2013). The EBMG accreditation ensures that graduates with an extra 2 years of clinical experience are eligible to gain professional registration with the EBMG, allowing them to work internationally (Paneque et al. 2017). The EBMG registration of graduates is strongly encouraged to sustain international professional standards and to embed them in an international community of GCs. Conversely, EBMG registration should also enable individuals trained in Europe to practice in the D‐A‐CH countries (Schwaninger et al. 2021).

#### 1.3.3 Legal Prerequisites for Genetic Counselors in the D‐A‐CH Countries

In Germany, according to § 7 (3) of the Genetic Diagnostics Act (GenDG, Gendiagnostikgesetz 2010), only qualified physicians are permitted to provide genetic counseling. This physician prerequisite includes a provision allowing non‐physician experts to be involved in counseling with the consent of the individuals concerned. In Austria, according to § 69 (2) of the Genetic Engineering Act (GTG, Gentechnikgesetz 2018), a similar physician prerequisite exists that includes a provision allowing the involvement of non‐medical individuals. The paragraph states that “the advisability of additional non‐medical counseling by a psychotherapist or social worker should be recommended”. While GCs are not explicitly mentioned, this is probably due to the absence of the profession in Austria and Germany when the laws were drafted. The GTG is currently under revision by the Austrian Ministry of Health involving an expert committee. In Switzerland, a full revision of the Federal Act on Human Genetic Testing was released in 2018, replacing the physician requirement with the term “qualified person” in Article 21 (GUMG 2018) which includes GCs.

#### 1.3.4 Status of the Recognition Process for the Genetic Counselor Profession in Switzerland

In Switzerland, formal recognition as a medical profession for GCs is not currently possible due to the lack of a Swiss‐based MSc program, but GCs can still pursue recognition through private law. The Swiss Federal Act on Human Genetic Testing allows a “qualified person” to provide genetic counseling (GUMG 2018). This designation permits GCs to work in collaboration and under the legal oversight of MGs within clinical settings. Consistent with this model, services performed by GCs under medical supervision cannot currently be billed separately and are instead covered by doctors' fees. Parallel initiatives led by the Swiss Medical Association (FMH) and supported by the Swiss Society of Medical Genetics (SGMG) aim to progress recognition of non‐physician health professions, including GCs, and to define a clear scope of practice. Also, the ASCG works collaboratively towards the recognition of the profession in all parts of Switzerland.

#### 1.3.5 How Can We Afford Genetic Counselors?

Currently, services performed by GCs under medical supervision cannot be billed separately and are instead covered by doctors' fees. In Germany, outpatient reimbursement is governed by the “Einheitlicher Bewertungsmaßstab” (EBM) that provides a billing code for each billable item; inclusion of new services typically follows assessment and approval by bodies such as the “Gemeinsamer Bundesausschuss” (GBA). If GCs are recognized as a distinct, academically trained healthcare profession, one proposed solution would be to allow for their own billing codes (e.g., to include them in the EBM). To justify the necessity and benefits of these services, innovation fund projects could be utilized to demonstrate healthcare needs and outcomes. The data collected from these projects could serve as a basis for assessment and approval by institutions like the GBA in Germany, which would then require health insurers to allocate funding for these services. These ideas highlight that the increasing demand for services, driven by new technologies and exacerbated by a shortage of skilled professionals, is resulting in an emerging gap in care. At the same time, funders in most countries are attempting to limit the financing of new services and the scope of analyses. Considering these challenges, the need for genetic counseling must be supported by ongoing professional development and the necessity to provide adequate patient care. Requesting specific billing numbers for GCs requires a clearly defined and jointly agreed SoP, including tasks that can be delegated from MGs to GCs. Furthermore, questions of professional liability and legal support need to be addressed in a concerted approach.

#### 1.3.6 GCs in the USA—What Can We Learn for the German‐Speaking World?

Regarding the long‐standing history of GCs in the US, this experience might promote the development of new strategies for the D‐A‐CH countries and motivate to strive for higher goals in the GC community. In the US, the first university‐trained GCs graduated from Sarah Lawrence College in New York in 1971, exactly 50 years prior to the first GCs educated in the D‐A‐CH countries. The profession has grown rapidly, from 427 GCs in 1982 to approximately 7000 in 2024, with a 100% growth rate in the last decade and similar projected development in the next 10 years (Ormond et al. 2023; NSCG 2024). Prerequisites and training of GCs in Europe and the US are similar, though certification is granted through a board exam in the US rather than a 2‐year trainee phase and portfolio registration in Europe. As of 2024, 35 US states offer licensure, with 12 more in progress, allowing GCs to bill insurances directly. GCs in the US commonly specialize in adult cancer (41%), prenatal (25%), and pediatric (23%) genetic counseling, working in various medical settings. About two‐thirds (63%) of US GCs actively participate in research and 51% co‐authored publications in 2023. The profession boasts high job satisfaction and demand, with salaries increased by 31% since 2016. With approximately 1240 certified MGs in the US in 2020, the ratio of MGs to GCs is approximately 1:5, reducing patients' waiting times for appointments significantly. Over half of GCs can offer appointments for new patients within 2 weeks, 21% see new patients within 1–3 days and 7% can offer same day appointments (Abacan et al. 2019; Quinn and Mazur 2022; NSCG 2024). This presentation highlighted the potential benefits and showcased the complementary role of GCs in addressing the increasing demand for genetic services. Concluding that GCs provide a comprehensive, collaborative approach to healthcare, complementing rather than replacing MGs (Schaaf 2021).

### 1.4 Session 2: Discussion on the Priorities for the GfH GC Commission

The workshop participants represented a broad group of stakeholders, including MGs, GCs, GC students from 31 university and private genetic centers across Germany, Austria, and Switzerland, allowing insights and input from various perspectives and viewpoints. Participants expressed a growing acceptance and demand for GCs in the D‐A‐CH countries, as well as challenges that need to be addressed by the newly founded GfH GC commission. Particularly, the discussion points about an efficient integration of GCs were in great coherence with some of the predefined goals of the GfH GC commission:

- A clear SoP based on legislature in D‐A‐CH and competency standards
- Definition of the professional profile of GCs within the multiprofessional team
- Remuneration, payment scales, and positions for GCs
- Billing of GC services towards insurances
- Legal requirements and recognition of GCs in the D‐A‐CH area.

With the GC profession entering the multiprofessional genetics teams, potential competency disputes among the three genetics professions need to be avoided (Paneque et al. 2022).

Session 2 was therefore designed to translate the commission's predefined aims—legal recognition and effective integration of GCs, development of a SoP, and strategies for billing and remuneration—into shared, near‐term priorities for the GfH GC commission. The session took place as a moderated plenary discussion with tracked participation and was documented in a structured written report for later analysis of the discussed content. To encourage open exchange, only the presentations but not the discussion were video recorded. Moderators synthesized contributions during the interactive discussion and reflected interim summaries back to the group to check shared understanding before documenting the agreed priorities. This discussion‐based process yielded broad agreement that defining a regionally aligned SoP is the most critical first task for the GfH GC commission.

It was discussed that the SoP should closely correspond with similar approaches in other European countries, for example, the ‘Scope of professional roles for genetic counselors and clinical geneticists in the UK’ (Middleton et al. 2023) as well as research on the tasks GCs perform internationally (Ormond et al. 2023). There was large agreement among participants that while international documents need to be the basis of the GC SoP, it also needs to respect the legal prerequisites of the D‐A‐CH countries. Particularly those workshop participants who already employ GCs or offer GC student placements requested a detailed list of tasks that they could refer to.

Several other advantages of a robust SoP were identified during the workshop. A clear definition of what GCs can provide for patients and how they can best be integrated within multidisciplinary teams will promote acceptance and appreciation by MGs and other members of the multiprofessional team (Catapano et al. 2022). A SoP could serve as the basis for employers to present the value of GCs to their Human Resources departments. Likewise, remuneration concepts and positions for GCs will hopefully be created more effectively when a SoP is in place. The clear definition of tasks delegable to GCs is also the basis for GC remuneration and the definition of billing numbers.

It was pointed out that the SoP could serve as the basis for further negotiations and implementation work for the profession, particularly if all three D‐A‐CH genetic societies and GC representatives could agree on such a document. A task that will require substantial goodwill and negotiations. Finally, only a definition with a clear profile will stand a chance to negotiate legal recognition, a task that will need to be approached both at the national and EU level.

## 2 Conclusion

The GfH is dedicated to human genetics in research, education, and patient care. To achieve this, the GfH has established various commissions focused on developing and organizing specific areas within the field. Currently, one of the GfH's initiatives is the integration of GCs into the provision of genetic services. This effort is based on the recognition that GCs possess unique core competencies that complement the expertise of medical and laboratory geneticists, fostering multiprofessional collaboration and patient care. In line with this goal, the GfH members have voted 86% in favor of the creation of a new GfH GC commission. The primary objective of supporting the GC profession in the D‐A‐CH region is to enhance access to genetic counseling by incorporating GCs into existing clinical genetic services. Workshop participants agreed that probably the most crucial first step in this integration process is the definition of a SoP that is supported broadly by genetics societies and GCs in the D‐A‐CH countries and will serve as the basis for all following steps of the GfH GC commissions' efforts.

## Author Contributions

Conceptualization: Gunda Schwaninger, Simone Heidemann, Sabine Rudnik‐Schöneborn, Johannes Zschocke. Methodology: Gunda Schwaninger. Formal analysis: Gunda Schwaninger, Kathrin Taxer. Visualization: Gunda Schwaninger. Writing – Original Draft: Gunda Schwaninger. Writing – Review and Editing: Gunda Schwaninger, Simone Heidemann, Kathrin Taxer, Sabine Rudnik‐Schöneborn, Johannes Zschocke. Revision: Gunda Schwaninger. Data curation: Gunda Schwaninger, Kathrin Taxer. Recourses: Johannes Zschocke.

## Funding

The authors have nothing to report.

## Disclosure

Declaration of Generative AI and AI‐Assisted Technologies in the Writing Process: During the preparation of this work, the first author used Perplexity.ai to check for language, spelling, and grammar. After using this tool, the first author reviewed and edited the content as needed and takes full responsibility for the content of the publication.

## Ethics Statement

All data presented was retrieved from a public online workshop. Speakers and participants were made aware that the presentations and discussion were recorded/documented for further use in a scientific publication. The research did not qualify for submission to an IRB or REC in the researchers' jurisdiction.

## Consent

Patient consent statement, permission to reproduce material from other sources, clinical trial registration.

## Conflicts of Interest

Author Sabine Rudnik‐Schöneborn declares that she has no conflicts of interest. Author Gunda Schwaninger, Simone Heidemann, Kathrin Taxer, and Johannes Zschocke declare that they are members of the GfH GC commission.

## Supporting information


**Figure S1:** Program of the Workshop: GfH Commission Genetic Counselors—Perspectives and Goals.

## Data Availability

Authors provide data availability and declare the absence of shared data. The corresponding author confirms that she has full access to all the data in the study and takes responsibility for the integrity of the data and the accuracy of the data analysis. All of the authors gave final approval of this version to be published and agree to be accountable for all aspects of the work in ensuring that questions related to the accuracy or integrity of any part of the work are appropriately investigated and resolved.
