# Supplementary material for: Strategic Development of the Genetic Counselor Profession in Germany, Austria, and Switzerland: The Establishment of the GfH Genetic Counselor Commission
Source: J Genet Couns. 2026 Jul 28;35(4):e70272. doi: 10.1002/jgc4.70272 (PMC13411196; doi:10.1002/jgc4.70272)
Supplement: Supplementary file 1 — Figure S1: Program of the Workshop: GfH Commission Genetic Counselors—Perspectives and Goals. [file JGC4-35-0-s001.docx]

| **Textbox 1.** Aims of the GfH GC commission integrated in its bylaws: |
| --- |
| **Leading initiatives** to work with the GfH board to create the conditions for the legal recognition of the new academic profession of GCs in the D-A-CH countries, defining a scope of practice (SoP) and promoting its integration into existing clinical genetic services. |
| **Developing strategies** for the billing and remuneration of GCs in close collaboration with the BVDH. |
| **Representing the profession** of GCs both within and outside of the GfH. Promoting and coordinating collaboration with national and international professional societies and interest groups for GCs; particularly the Austrian Society of Human Genetics (ÖHG), Swiss Society of Medical Genetics (SGMG), and the Association Suisse des Conseillers en Génétique (ASCG) as well as the European Board of Medical Genetics (EBMG) as the official registration body for European GCs. |
| **Advancing the profession** in GC education, continuing professional development, supervision, and research. |

**Supplementary Figure 1.**

Program of the Workshop: GfH Commission Genetic Counselors - Perspectives and Goals.

**
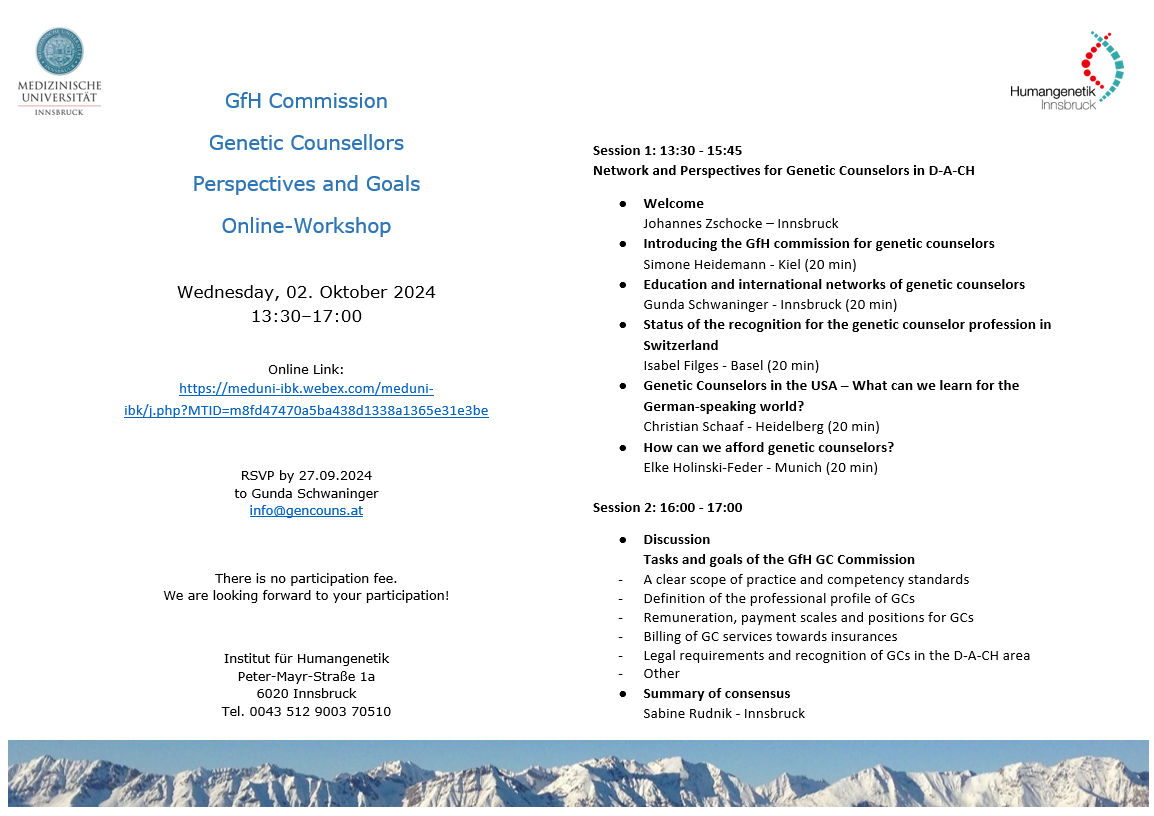
**

**References**

Abacan M, Alsubaie L, Barlow-Stewart K, Caanen B, Cordier C, Courtney E, Davoine E, Edwards J, Elackatt NJ, Gardiner K, Guan Y, Huang LH, Malmgren CI, Kejriwal S, Kim HJ, Lambert D, Lantigua-Cruz PA, Lee JMH, Lodahl M, Lunde Å, Macaulay S, Macciocca I, Margarit S, Middleton A, Moldovan R, Ngeow J, Obregon-Tito AJ, Ormond KE, Paneque M, Powell K, Sanghavi K, Scotcher D, Scott J, Juhé CS, Shkedi-Rafid S, Wessels TM, Yoon SY, Wicklund C. (2019). The Global State of the Genetic Counseling Profession. Eur J Hum Genet. 2019 Feb;27(2):183-197. doi: 10.1038/s41431-018-0252-x

Catapano, F., El Hachmi, M., Ketterer-Heng, N., Renieri, A., Mari, F., Morris, M. and Cordier, C. (2022) 'The role of the Genetic Counsellor in the multidisciplinary team: the perception of geneticists in Europe', Eur J Hum Genet. 2022 Dec;30(12):1432-1438. doi: 10.1038/s41431-022-01189-5

Gendiagnostikgesetz (2010). GenDG, Fassung vom 16.05.2024. Retrieved 13/01/2026 from <https://www.gesetze-im-internet.de/gendg/inhalts_bersicht.html>

Gentechnikgesetz (2018). GTG, Fassung vom 21.02.2018. In: Austrian Ministry of Health, ed. BGBl. Nr. 510/1994. Bundesministerium für Arbeit, Soziales, Gesundheit und Konsumentenschutz (Austrian Ministry of Health). Retrieved 13/01/2026 from <https://www.ris.bka.gv.at/>

GUMG (2018). Bundesgesetz über genetische Untersuchungen beim Menschen (GUMG). Retrieved 13/01/2026 from <https://www.fedlex.admin.ch/eli/cc/2022/537/de>

Middleton, A., Taverner, N., Houghton, C., Smithson, S., Balasubramanian, M. and Elmslie, F. (2023) 'Scope of professional roles for genetic counsellors and clinical geneticists in the United Kingdom : Position on behalf of the Association of Genetic Nurses and Counsellors and the Clinical Genetics Society', Eur J Hum Genet. 2023 Jan; 31(1):9-12. doi: 10.1038/s41431-022-01214-7

NSGC (2024). Professional Status Survey. Retrieved 13/01/2026 from <https://www.nsgc.org/Portals/0/Docs/Policy/PSS%202024%20Executive%20Summary_Final.pdf?ver=xMyKy1O8H749GCBpxy6NiA%3d%3d>

Ormond, K. E., Hayward, L., Wessels, T.-M., Patch, C. and Weil, J. (2023) 'International genetic counseling: What do genetic counselors actually do?', J Genet Couns. 2024 Apr;33(2):382-391. doi: 10.1002/jgc4.1735

Paneque, M. , Moldovan, R. , Cordier, C. , Serra‐Juhe, C. , Feroce, I. , Pasalodos, S. , & Skirton, H. (2017). The perceived impact of the European registration system for genetic counsellors and nurses. European Journal of Human Genetics, 25(9), 1075–1077. 10.1038/ejhg.2017.84

Paneque, M., Liehr, T., Serra Juhé, C., Moog, U., Melegh, B. and Carreira, I. (2022) 'The need for recognition of core professional groups in genetics healthcare services in Europe', Eur J Hum Genet, 30, pp. 640 <https://doi.org/10.1002/ajmg.c.31607>

Quinn, E., Mazur, K. (2022). The experiences of UK-based genetic counsellors working in mainstream settings. Eur J Hum Genet 30, 1283–1287 (2022). https://doi.org/10.1038/s41431-022-01158-y

Schaaf, C. P. (2021). "Genetic counseling and the role of genetic counselors in the United States" Medizinische Genetik, vol. 33, no. 1, 2021, pp. 29-34. <https://doi.org/10.1515/medgen-2021-2054>

Schwaninger G, Heidemann S, Hofmann W, Maurer T, Mayerhanser K, Ronez J, et al. (2021). Prospects and challenges for the genetic counsellor profession in the German-speaking countries: report of a workshop. MedGen 2021; 33(1): 35–44. doi: 10.1515/medgen-2021-2055

Skirton, H. , Barnoy, S. , Ingvoldstad, C. , Kessel, I. V. , Patch, C. , O'Connor, A. , Serra‐Juhe, C. , Stayner, B. , & Voelckel, M.‐A. (2013). A Delphi study to determine the European core curriculum for Master programmes in genetic counselling. European Journal of Human Genetics, 21(10), 1060–1066. 10.1038/ejhg.2012.302
